# A Dynamic Compensation Method Based on Pulse Width for Laser Ranging and Distance Determination in Precision-Guided Aircraft

**DOI:** 10.3390/mi16121409

**Published:** 2025-12-15

**Authors:** Jinghao Li, Zhipeng Li, Yuheng He, Kuizheng Li, Hejuan Chen

**Affiliations:** 1 School of Mechanical Engineering, Nanjing University of Science and Technology, Nanjing 210094, China; leejh1997@njust.edu.cn (J.L.);; 2 School of Mechanical Engineering, Jiangsu Ocean University, Lianyungang 222005, China; 2021000138@jou.edu.cn

**Keywords:** precision-guided aircraft, initiation device, laser ranging, pulse width, dynamic compensation, distance correction

## Abstract

This paper proposes a dynamic compensation method for laser ranging based on pulse width for the miniaturization and high-precision requirements of the initiation device in precision-guided aircraft. The study aims to improve the measurement accuracy of the laser ranging unit in the initiation device system and ensure the accuracy and reliability of its fixed-distance initiation decision. The variation in echo pulse width is analyzed by studying laser echo characteristics. The pulse width and the detection distance exhibit an approximately linear negative correlation within the middle range of the applicable distance range. A dynamic compensation method is proposed based on a dual-correction approach using a static lookup table and dynamic compensation. This method establishes the mapping relationship between pulse width and distance deviation, and achieves distance correction by adding distance deviation compensation to the basic value from the static lookup table. The dynamic compensation system integrated with calibration and correction is designed and implemented, and the feasibility of the dynamic compensation method is verified by testing. The relative error between the calculated correction distance and the actual distance is small, and the average relative error is about 1.33%. The proposed method provides key technical support for the establishment of miniaturized and intelligent initiation devices.

## 1. Introduction

The initiation device is a control device that utilizes target and environmental information to detonate or ignite the warhead under predetermined conditions [1]. It serves as a core component in missiles and other precision-guided aircraft. During a missile–target encounter, when the target enters the effective zone of the initiation device and reaches a preset distance range, the detonation of the warhead by the initiation device is known as initiation at a fixed distance. On the other hand, detonating the warhead at a preset time is known as initiation at a fixed time. These two initiation methods are common in mechanical initiation devices, electromechanical initiation devices, and proximity initiation devices [2,3,4]. Although initiation at a fixed time offers precise time control, the actual explosion point can be affected by variations in the projectile flight velocity. Initiation at a fixed distance achieves distance measurement through methods such as counting projectile rotations, using a mechanical probe, or employing laser-based proximity detection. This initiation method is not affected by muzzle velocity and can achieve more accurate distance control. Therefore, initiation at a fixed distance provides superior precision in distance control compared to initiation at a fixed time.

In an initiation device system, laser-based proximity detection uses lasers to sense and assess the status, distance, and even characteristics of nearby targets [5,6,7]. It is designed to accurately measure or determine one or several specific distances, rather than continuously measuring all distance values within a range. Therefore, laser-based proximity detection in an initiation device is a process of judgment and decision making, with the core being to determine whether the target has entered a preset proximity distance. Mechanical fixed-distance initiation devices, such as pop-out probes, have the advantages of relatively simple structure and high reliability. However, they perform poorly in terms of automatic distance adjustment. Therefore, laser-based proximity devices are being increasingly adopted. These devices are designed and manufactured according to the specific requirements of the initiation device, and they must meet the design specifications and comply with standards.

Laser ranging accuracy is one of the main technical indicators of laser-based proximity devices [8,9]. Existing methods for improving laser ranging accuracy primarily follow two technical pathways. One is the processing pathway based on signal amplitude, such as multithreshold time discrimination [10] and adaptive threshold time discrimination [11]. This class of methods suppresses amplitude fluctuations through analog circuits or logarithmic amplification. Their core contribution lies in achieving an amplitude-interference-resistant stable timing point, ingeniously solving the when-to-trigger problem caused by variations in target reflectivity. However, their essence does not address the echo pulse waveform distortion caused by hardware individual differences in laser diodes and photodetectors. This systematic deviation at the waveform level is a deeper-rooted cause limiting inter-device consistency and absolute accuracy. The second pathway is the algorithmic pathway based on complex signal processing, such as the wavelet denoising method [12], BP neural network algorithm [13], second-degree polynomial approximation method [14], and pseudo-random code modulation [15]. These methods theoretically possess powerful feature extraction and noise suppression capabilities. However, their implementation relies on high computational costs, large training datasets, or complex parameter tuning. The above methods are mostly applied to time-of-flight laser ranging based on the pulsed laser. In addition, there are also laser ranging methods that use other forms of lasers. such as the self-mixing interferometry method [16], dispersion interferometry method [17], range-gated active imaging method [18], coherent frequency modulation continuous wave method [19], etc. However, these methods are mostly aimed at high-precision surveying or industrial inspection, and their applicability to low-cost, embedded control devices is limited. Therefore, these limitations render the above methods less ideal for high-speed, high-reliability applications such as the initiation device in precision-guided aircraft, where deterministic performance and minimal latency are paramount.

To overcome the aforementioned limitations, this paper proposes a dynamic compensation method based on pulse width time domain features. This method explicitly define the echo pulse width as a feature code capable of characterizing the inherent systematic error of the hardware. By establishing the compensation model between the pulse width and distance deviation, we achieve a real-time correction distance that requires no extensive training and only simple arithmetic operations. This study aims to improve the measurement accuracy of the laser ranging unit in the initiation device system and ensure the accuracy and reliability of its fixed-distance initiation decision. It will provide key technology support for the application of high-performance, high-integration microsystems in precision guidance.

## 2. Principle of Dynamic Compensation Based on Pulse Width

### 2.1. Overall Architecture of Dynamic Compensation System

**Figure 1:**
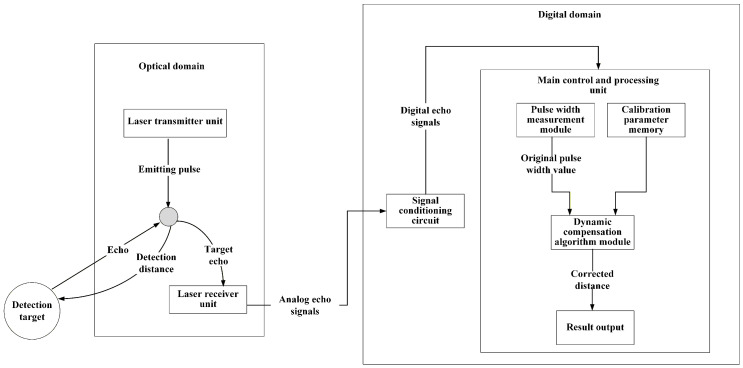
The overall architecture of the dynamic compensation system.

The overall architecture of the dynamic compensation system is shown in Figure 1. The system is composed of an optical domain and a digital domain. The optical domain includes a laser transmitter unit and laser receiver unit, which outputs analog echo signals to the digital domain. The digital domain includes a signal conditioning circuit, and a main control and processing unit serving as the system core. The signal conditioning circuit converts the analog echo signals into digital echo signals. The main control and processing unit contains a pulse width measurement module, calibration parameter memory and a dynamic compensation algorithm module.

**Figure 2:**
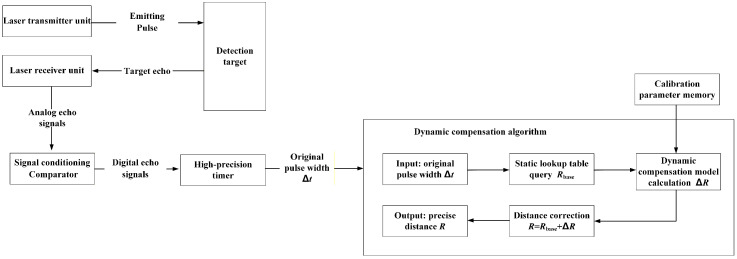
The design scheme for pulse width measurement and dynamic compensation.

Figure 2 shows the design scheme for pulse width measurement and dynamic compensation. The key to dynamic compensation lies in determining the relationship between the pulse width and distance deviation. The principle of the dynamic compensation method based on pulse width is as follows: The laser transmitter unit emits a pulse, which is reflected by the target and converted into electrical analog signals by the laser receiver unit. These signals are then converted into digital pulses through signal conditioning and a comparator. The original pulse width is measured using the high-precision timer. The basic distance corresponding to the pulse width is obtained by querying the static lookup table. The distance deviation is calculated by the dynamic compensation model with the pre-stored calibration parameters. The precise distance is corrected and output by adding the base distance and the distance deviation.

### 2.2. Relationship Between Echo Pulse Width and Detection Distance

**Figure 3:**
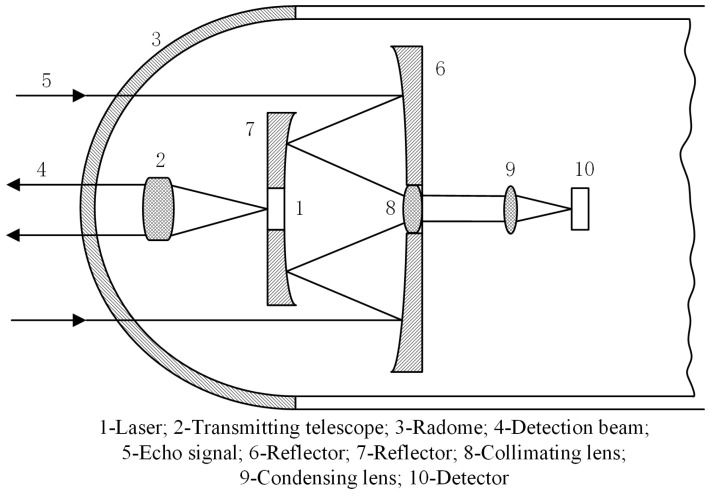
Schematic diagram of optical path of laser detection system in initiation device.

The laser detection system in an initiation device with perfect performance is a complete small laser radar, which has the functions of speed, direction, and distance measurement. The laser detection system is mainly composed of two parts: the transmitter and the receiver. The transmitter system generally includes circuits for signal generation and processing, as well as the transmitting optical system. It forms the laser beam with the required detection field of view. The receiver system comprises the receiving optical system, the detector–preamp signal processing circuit, and the control and execution circuit. The field of view of the receiving optical system is matched to that of the transmitting optical system. Currently, an active laser detection system is widely used. The transmitting and receiving optical systems often share the coaxial optical path, as shown in Figure 3. This structure can reduce the overall initiation device size and increase the effective receiving area. In Figure 3, the echo signal “5” of the laser detection system passes through reflectors “6” and “7”, then travels through the collimating lens “8” and condensing lens “9” before reaching the detector “10”. The detector is a critical component of the detection system. Its performance directly impacts the overall performance of the initiation device system. Currently, PIN silicon photodiodes are widely used as detectors due to high sensitivity, fast response time, and low equivalent noise power.

The detection distance is not only related to the performance of receiving transmitting systems, but also related to the target characteristics and background conditions. Its distance sharp cut-off characteristic is ensured by distance accuracy. Distance accuracy is a very important index of the initiation device. In general, the distance accuracy is mainly determined by the rise time of the laser pulse. Depending on the target illuminated by the laser, the amplitude of the reflected echo signal at the same distance can vary by several orders of magnitude due to differences in engagement attitudes. Therefore, the largest absolute distance error is the distance error resulting from the laser pulse rise time.

Assuming the target exhibits diffuse reflection characteristics for the laser waves, the received laser power can be expressed as follows:

(1) $$P_{r}=\frac{P_{t}G_{t}}{4\pi R_{t}^{2}}\frac{\sigma }{4\pi R_{r}^{2}}\frac{\pi D^{2}}{4}\eta_{\mathrm{atm}}^{2}\eta_{\mathrm{sys}}$$

where $P_{t}$ is the transmitted pulsed laser power; $G_{t}$ is the gain of the transmitting optical system; $R_{t}$ is the distance between the laser transmitting system and the target; $R_{r}$ is the distance between the laser receiving system and the target; in this paper, $R_{t}=R_{r}=R$; σ is the laser scattering cross-section; *D* is the diameter of the receiving system; $\eta_{\mathrm{atm}}$ is the atmospheric transmission coefficient; and $\eta_{\mathrm{sys}}$ is the optical transmission coefficient of the laser detection system.

The first term on the right side of Equation (1) represents the illuminance of the pulsed laser emission surface. The spatial distribution characteristics of the laser pulse describe the spatial energy distribution of the beam perpendicular to its propagation direction. The spatial mode field distribution of the laser is TEM00 (Transverse Electromagnetic Mode 00) and its light intensity distribution obeys the Gaussian distribution. The laser beam propagates along the z-axis direction and the expression of beam illuminance is as follows:

(2) $$E\left(x,y,z\right)=g\left(x,y,z\right)P_{t}=\frac{2P_{t}}{\pi \omega^{2}}\exp\left[-2\left(\frac{x^{2}+y^{2}}{\omega^{2}}\right)\right]$$

where ω is the spot radius of the Gaussian beam at distance *z*, given by $\omega =\omega_{0}\sqrt{1+{\left(\lambda z/\pi \omega_{0}^{2}\right)}^{2}}$; λ is the wavelength of the pulsed laser; $\omega_{0}$ is the beam waist radius at the source, defined as $\omega_{0}=2\lambda /\pi \phi$; and ϕ is the full angle of beam divergence.

The output power of the pulsed laser is a function related to time. Let the output pulse be Gaussian and its function expression be as follows:

(3) $$P_{t}\left(t\right)=P_{0}\exp\left[-{\left(\frac{t}{\tau }\right)}^{2}\right]$$

where $P_{0}$ is the peak power of the laser pulse and τ is the laser pulse width, defined as the half-width of the Gaussian pulse at 1/e of its peak intensity.

The equation for the scattering cross-section per unit element of laser scattering is as follows:

(4) $$d\sigma =4\pi f_{r}\left(\alpha \right)\cos^{2}\alpha dS$$

where $f_{r}\left(\alpha \right)$ is the bidirectional reflectance distribution function (BRDF) of the unit surface element; dS is the unit surface element of laser scattering, with cosαdS=dxdy; and α is the angle of incidence.

The echo pulse signal received by the pulsed laser detection system can be regarded as the temporal superposition of echoes scattered from various points on the target surface. The echo pulse power received by the detection system is as follows:

(5) $$P_{r}\left(t,R\right)=\frac{\pi D^{2}}{4R^{2}}\eta_{\mathrm{atm}}^{2}\eta_{\mathrm{sys}}\;\int \int \;g\left(x,y,R\right)P_{t}\left(t-\frac{2R+2x\tan\alpha }{c}\right)f_{r}\left(\alpha \right)\cos\alpha dxdy$$

In proximity detection, the target can be regarded as an extended target, meaning the laser spot on the target is a circle with a radius of ω, within which approximately 95.45% of the total laser energy is concentrated. The integral range can be extended to infinity during calculation and then the echo pulse power becomes the following:

(6) $$P_{r}\left(t,R\right)=\frac{P_{0}D^{2}f_{r}\left(\alpha \right)\cos\alpha }{2\omega^{2}R^{2}}\eta_{\mathrm{atm}}^{2}\eta_{\mathrm{sys}}\int_{-\infty }^{\infty }e^{-2\frac{y^{2}}{\omega^{2}}}dy\int_{-\infty }^{\infty }e^{-2\frac{x^{2}}{\omega^{2}}}e^{\frac{-{\left(t-\frac{2R+2x\tan\alpha }{c}\right)}^{2}}{\tau^{2}}}dx$$

Performing the integration with respect to *x* and *y*, respectively, the laser echo pulse power under vertical illumination of the target is obtained as follows:

(7) $$P_{r}=\frac{\pi P_{0}D^{2}f_{r}}{4R^{2}}\eta_{\mathrm{atm}}^{2}\eta_{\mathrm{sys}}e^{\frac{-{\left(t-t_{r}\right)}^{2}}{\tau^{2}}}$$

where $t_{r}=2R/c$, with *c* being the speed of light, and $f_{r}$ is the bidirectional reflection coefficient under vertical illumination, with α=0. For the Lambertian target, $f_{r}=\rho /\pi$, with ρ being the target reflectivity.

From Equation (7), different target characteristics and distances can lead to drastic changes in echo signal amplitude (up to 100 dB), resulting in great laser ranging error when using the fixed-threshold method. Therefore, the laser detection system is required to have the ability of instantaneous amplitude control. To address this, the half-maximum detection technique is commonly employed to regulate echo amplitude, thereby improving ranging accuracy. Half-maximum detection is a classic time-point normalized instantaneous signal processing method. By utilizing logarithmic amplification and a comparator, the timing trigger is always activated at the same relative position on the echo pulse waveform, independently of the absolute amplitude.

**Figure 4:**
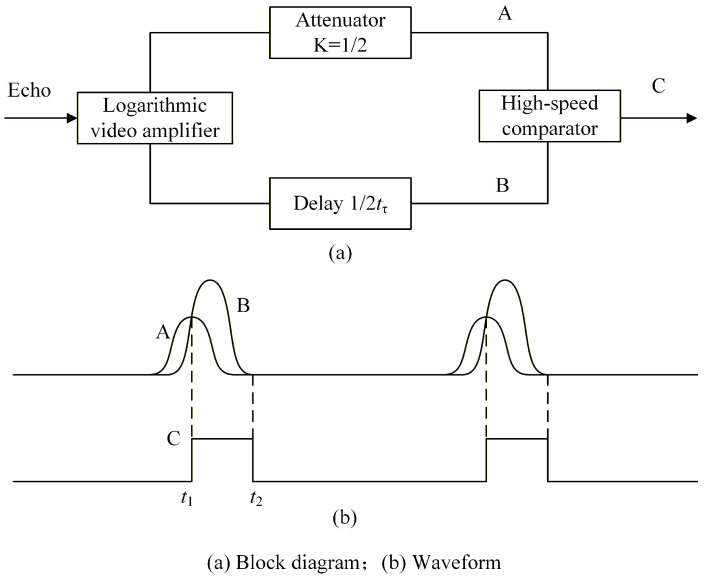
Half-maximum detection method.

Figure 4 is the schematic diagram of the half-maximum detection method. The amplitude range of the echo signal is greatly reduced after passing through the logarithmic video amplifier. The amplitude-halved signal A (a static image signal) and the signal B delayed by half the rise time $t_{\tau }$ are fed into the high-speed comparator, generating the constant-amplitude output signal C.

The echo signal and the constant-amplitude signal C are, respectively, denoted as $V\left(t\right)$ and $V_{C}$. In Figure 4, signal C is triggered at the intersection point of waveforms A and B (at time $t_{1}$). Regardless of variations in the amplitude of $V\left(t\right)$, the comparator is consistently triggered at the fixed time $t_{1}$ and the C pulse drops at time $t_{2}$ when signal B ends. Therefore, the pulse width Δt of the C pulse signal obtained can be expressed as follows:

(8) $$\Delta t=t_{2}-t_{1}$$

The echo signal represented by $V\left(t\right)$ is an analog signal. The voltage expression is as follows:

(9) $$V\left(t\right)=\frac{ℜM\rho P_{0}D^{2}}{4R^{2}}\eta_{\mathrm{atm}}^{2}\eta_{\mathrm{sys}}e^{\frac{-{\left(t-t_{r}\right)}^{2}}{\tau^{2}}}=A_{r}e^{\frac{-{\left(t-t_{r}\right)}^{2}}{\tau^{2}}}$$

where *ℜ* is the detector responsivity; *M* is the amplification factor of the receiving circuit; and ρ is the target reflectivity.

The actual laser pulse may deviate slightly from the ideal Gaussian shape but, within the bandwidth of this system, its rising and falling edges have good symmetry, making the Gaussian assumption a valid approximation. Due to the compromise between the system noise floor and signal amplitude, when the amplitude of $V\left(t\right)$ is less than 0.1 V, the signal is considered negligible. Therefore, let $V\left(t\right)=\frac{A_{r}}{2}$ and V(t)=0.1, respectively, and obtain the following:

(10) $$t_{1}=t_{r}-\tau \sqrt{\ln2};t_{2}=t_{r}+\tau \sqrt{\ln\left(\frac{5ℜM\rho P_{0}D^{2}\eta_{\mathrm{atm}}^{2}\eta_{\mathrm{sys}}}{2R^{2}}\right)}$$

The expression for the relationship between the pulse width Δt of the laser echo signal and the detection distance *R* is as follows:

(11) $$\Delta t=\tau \sqrt{\ln2}+\tau \sqrt{\ln\left(\frac{5ℜM\rho P_{0}D^{2}\eta_{\mathrm{atm}}^{2}\eta_{\mathrm{sys}}}{2R^{2}}\right)}$$

**Figure 5:**
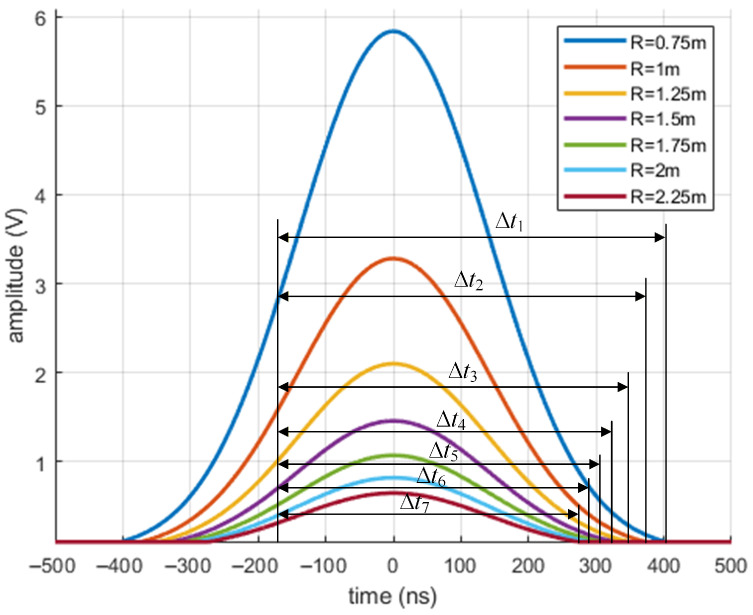
Echo signal curves at different distances *R*.

Assuming the target is the white-colored paper and the parameters of the laser ranging device have been designed as shown in Table 1, let the detection distances be R=0.75,1,1.25,1.5,1.75,2,2.25 m. The echo signals $V\left(t\right)$ at these different detection distances are calculated using Equation (9). The results in Figure 5 show the following:

(1) In Equation (9), $t_{r}$ represents the phase offset of the echo signal relative to the transmitted signal and does not affect the shape of the echo signal. Therefore, its influence is omitted in Figure 5.
(2) The amplitude of the echo signal $V\left(t\right)$ decreases as the distance *R* increases. Calculations are performed at 0.25 m intervals. When *R* increases from 0.75 m to 2.25 m, the maximum amplitude of the echo signal drops from 5.9 V to 0.65 V, a decrease of approximately 89%.
(3) The pulse width Δt of the echo signal $V\left(t\right)$ narrows significantly as the distance *R* increases. When *R* increases from 0.75 m to 2.25 m, the pulse width Δt decreases from 570 ns to 440 ns, a reduction of approximately 23%.

These results indicate that the echo signal of the laser ranging device attenuates significantly with increasing detection distance, and the pulse width Δt of the echo signal changes noticeably as the distance increases.

**Figure 6:**
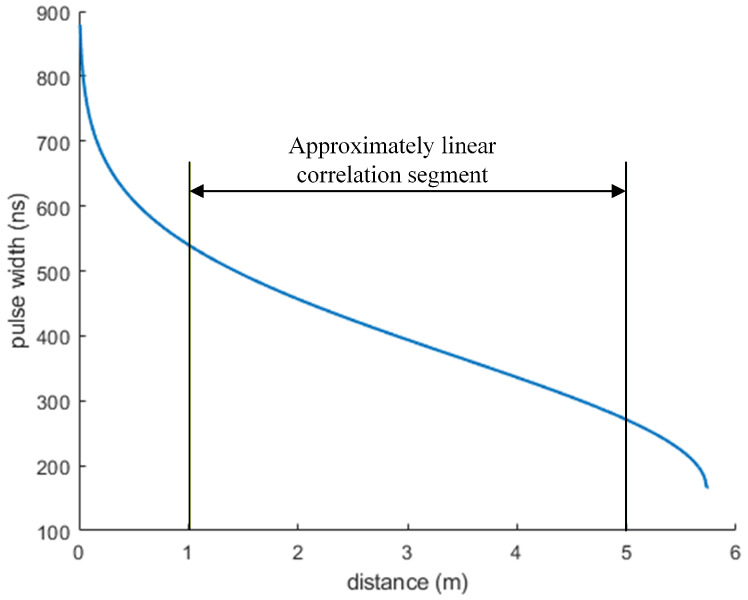
Relationship curve between Δt and *R*.

Figure 6 shows the relationship curve between Δt and *R*. It can be observed that Δt and *R* exhibit an inverse relationship. When *R* is within the range of 1 m to 5 m, Δt and *R* exhibit an approximately inverse linear relationship. However, when *R* is less than 1 m or greater than 5 m, a strongly nonlinear inverse relationship is observed between the two parameters. By adjusting the cut-off threshold of the echo pulse, the applicable range of the approximate linear region can be changed.

### 2.3. Dynamic Compensation Method

The most common method for ranging based on a pulsed laser is the time-of-flight method. Its principle is to measure the time interval between the rising edges of the transmitting and receiving pulses, and then multiply it by half the speed of light to determine the distance. Due to the high speed of light and the timing error of the timer itself, a time change of 1 ns can cause an error of approximately ±0.2 m [20]. Therefore, the time-of-flight method performs well at long distances, while it shows relatively large errors in short-range detection not exceeding 5 m. The existing methods for improving the ranging accuracy of the time-of-flight method mainly follow two technical pathways: the time discrimination pathway based on signal amplitude and the algorithm pathway based on complex signal processing. The former does not address the echo pulse waveform distortion caused by individual hardware differences in the laser diodes and photodetector, which is a deeper-rooted cause limiting inter-device consistency and absolute accuracy. The latter implementation relies on high computational costs, large training datasets, or complex parameter tuning. This makes them difficult to deploy in an initiation device system that emphasizes real-time performance, reliability, and low cost.

**Figure 7:**
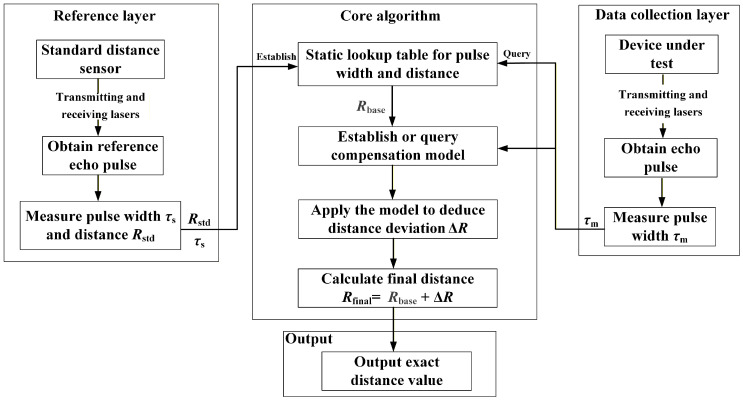
Block diagram of the dynamic compensation method.

The dynamic compensation method proposed in this article explicitly defines the echo pulse width as a feature code capable of characterizing the inherent systematic error of the hardware. By establishing the exclusive compensation model between the pulse width and distance deviation for each device, we achieve real-time distance correction that requires no extensive training and only simple arithmetic operations. The core of the method lies in the correction process based on the temporal feature of the echo pulse width. First, the static lookup table (LUT) is established for the device, providing a basic distance $R_{\mathrm{base}}$ from the original pulse width. Second, a dynamic device-specific compensation model is employed. This model uses the instantaneous pulse width to calculate a real-time compensation distance deviation value ΔR. Finally, the basic distance and distance deviation are added together to obtain the final distance. Dynamic compensation means that the deviation value needs to be calculated in real time for each measurement and then compensation is made for the basic value corresponding to the instantaneous pulse width to obtain the distance. This dynamic compensation method can be used alone for short-range distance measurement with a range of no more than 5 m or in conjunction with the time-of-flight method for mutual verification. Figure 7 is the block diagram of the dynamic compensation method that uses echo pulse width as the compensation information source.

The core principle and operational workflow of the dynamic compensation method are described below.

(1) Build the static lookup table.

The high-precision standard distance sensor is used to measure the target at various distances, obtaining a large set of distance $R_{\mathrm{std}}$ and pulse width $\tau_{s}$. These data are used to establish the static lookup table for pulse width and distance (LUT).

(2) Establish the compensation model.

The device under test measures the target at several specific distances $R_{m}$, recording the corresponding pulse widths $\tau_{m}$. For each $\tau_{m}$, the corresponding basic distance $R_{\mathrm{base}}$ is retrieved from the static lookup table.

(12) $$R_{\mathrm{base}}=LUT\left(\tau_{m}\right)$$

Then, the calculation formula of the detection distance deviation is as follows:

(13) $$\Delta R=R_{m}-R_{\mathrm{base}}$$

By performing multiple measurements at different distance points, the mathematical model describing this deviation can be established, expressed as follows:

(14) $$\Delta R=f\left(\tau_{m}\right)$$

Equation (14) is the established mathematical model of the distance deviation ΔR, which serves as the core model for dynamic compensation. This model reflects the relationship between the pulse width and distance deviation.

(3) Dynamic correction of distance result.

When measuring at an unknown distance, the device under test only needs to measure the echo pulse width $\tau_{m}$.

By querying through the internal algorithm and utilizing the pre-established compensation model, the current distance deviation ΔR is inversely derived. Finally, the corrected and more accurate distance value is obtained through the distance correction formula.

The distance correction formula is as follows:

(15) $$R_{\mathrm{final}}=R_{\mathrm{base}}+\Delta R$$

In Equation (15), $R_{\mathrm{base}}$ is a rough basic distance value without dynamic compensation obtained by querying the static lookup table for pulse width and distance (LUT). Therefore, the physical meaning of Equation (15) is that the final accurate distance is the sum of the basic distance value $R_{\mathrm{base}}$ and the distance deviation ΔR calculated from the pulse width.

This demonstrates that the essence of dynamic compensation lies in treating the pulse width as a characteristic code that reflects the performance state of the device under test. By comparing and decoding this code against the reference from the standard sensor, the distance information in the characteristic code is resolved. Through the process of processing, analysis, and decision making, an initiation command is finally output.

## 3. Design and Implementation of Dynamic Compensation System

### 3.1. Working Principle of Dynamic Compensation System

**Figure 8:**
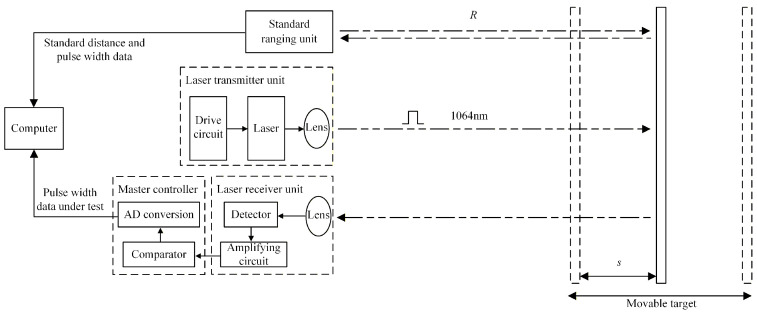
Working principle diagram of dynamic compensation system.

**Figure 9:**
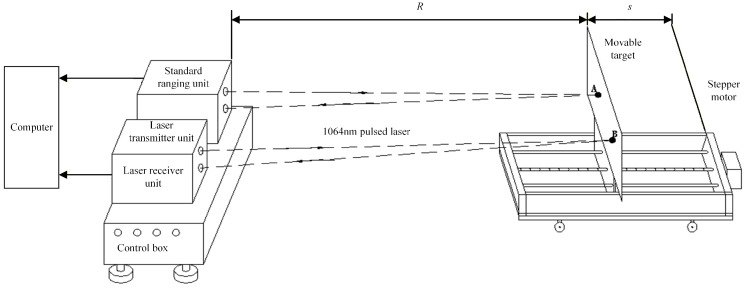
Composition of dynamic compensation system.

The system consists of a standard ranging unit, laser transmitter unit, laser receiver unit, master controller, core module, and output module. Figure 8 is the working principle diagram of the system designed based on the overall architecture in Figure 1 and Figure 2 (the core module and output module are not drawn in the diagram). The standard ranging unit is mainly the standard distance sensor system. The laser transmitter and receiver units form the optical system of the device under test and can be replaced. The master controller includes the signal conditioning circuit with comparator and the AD conversion module. The computer mainly includes the core module and the output module. The core module is the implementation of the dynamic compensation algorithm, used to calculate the correction distance. The output module displays the precise correction distance on the screen. Figure 9 is the system implementation of Figure 8.

### 3.2. Design and Implementation of Dynamic Compensation Algorithm

**Figure 10:**
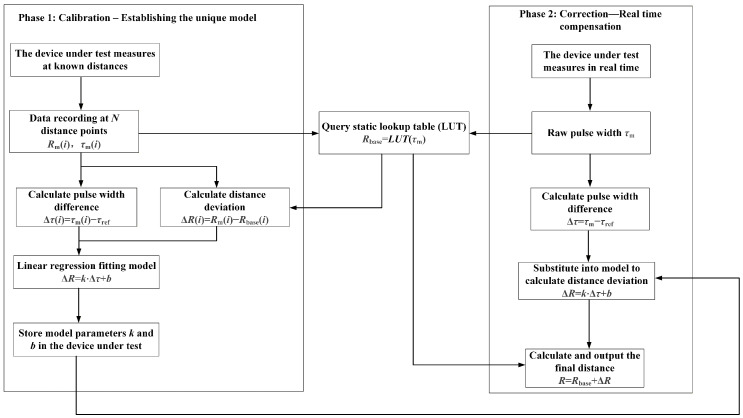
Dynamic compensation algorithm.

The dynamic compensation algorithm designed in this study is divided into two phases, calibration and correction, with the workflow illustrated in Figure 10.

The static lookup table for pulse width and distance (LUT) is established as the cornerstone of the dynamic compensation system, determining the accuracy of the basic distance value $R_{\mathrm{base}}$. At numerous distance points covering the full range of the device under test, the high-precision standard sensor measures and records the corresponding relationship between the pulse width $\tau_{s}$ and distance $R_{\mathrm{std}}$, forming the reference dataset. The LUT is constructed after outlier removal and smoothing pre-processing.

The device under test rapidly locates the interval of the currently measured $\tau_{m}$ within the LUT by using the binary search method and computes the corresponding basic distance value $R_{\mathrm{base}}$ in real time by using the linear interpolation method. This method ensures high precision while maintaining an efficient query performance.

(1) Calibration phase

The calibration phase aims to establish the unique pulse width and distance deviation relationship model for each device under test.

In a laboratory environment with controllable temperature and humidity, the static lookup table for pulse width and distance (LUT) is established for the device under test through the high-precision standard sensor (with accuracy one order of magnitude higher than the device under test). The LUT can be considered permanent and fixed. For any raw pulse width value $\tau_{m}$ measured by the device under test, the closest basic distance value $R_{\mathrm{base}}$ without dynamic compensation can be obtained by querying this LUT.

The device under test aims at the target in the same way and, at multiple known distance points $R_{m}\left(i\right)$ (*i* = 1, 2, …, *N*), the pulse widths $\tau_{m}\left(i\right)$ measured by the device are recorded. For the stability and efficiency of computer data processing, data centering is applied by subtracting a fixed constant $\tau_{\mathrm{ref}}$ from $\tau_{m}\left(i\right)$.

(16) $$\Delta \tau \left(i\right)=\tau_{m}\left(i\right)-\tau_{\mathrm{ref}}$$

The corresponding basic distance $R_{\mathrm{base}}\left(i\right)$ for $\tau_{m}\left(i\right)$ is retrieved by querying the static lookup table.

(17) $$R_{\mathrm{base}}\left(i\right)=LUT\left(\tau_{m}\left(i\right)\right)$$

Therefore, the distance deviation ΔR(i) corresponding to the pulse width difference Δτ(i) at each calibration point *i* is as follows:

(18) $$\Delta R\left(i\right)=R_{m}\left(i\right)-R_{\mathrm{base}}\left(i\right)$$

The data points {Δτ(i),ΔR(i)} are linearly regressed by using the least squares method and the compensation model is obtained as follows:

(19) ΔR=k·Δτ+b

In Equation (19), *k* and *b* represent the fitting proportional coefficient and zero offset, respectively. These parameters are programmed into the memory of the device under test for use during the correction phase.

(2) Correction phase

During the correction phase, for each new measurement, the device under test only needs three steps: measure, query, and calculate.

The device under test measures a pulse width $\tau_{m}$. By using the binary search and linear interpolation method, it queries the static lookup table to obtain $R_{\mathrm{base}}=LUT\left(\tau_{m}\right)$. Then, calculate $\Delta \tau =\tau_{m}-\tau_{\mathrm{ref}}$, substitute this into the compensation model to calculate ΔR, and output the final distance $R=R_{\mathrm{base}}+\Delta R$.

The algorithm has a small amount of calculation, and has certain requirements for data storage and reading. It can meet the requirements of the rapidity of the initiation device system and effectively ensure the real-time performance of the system.

## 4. Testing and Results

### 4.1. Establishment of Static Lookup Table (LUT)

In a laboratory environment with controllable temperature and humidity, the high-precision standard sensor was aimed at the target surface—a white-colored paper. Measurements of pulse width were taken at 0.1 m intervals over a distance range from 0.9 m to 3.6 m. At each distance $R_{\mathrm{std}}$, 20 measurements were performed and the average value of these 20 measurements was taken as the pulse width $\tau_{s}$ for that distance point. The data points $\left\{R_{\mathrm{std}},\tau_{s}\right\}$ were sorted in ascending order of distance to form the LUT, as shown in Table 2.

### 4.2. Establishment of Compensation Model for the Device Under Test

The device under test aimed at the target in the same way and the pulse width $\tau_{m}$ was measured at eleven distance points $R_{m}$. The corresponding basic distance $R_{\mathrm{base}}$ for each $\tau_{m}$ was obtained by querying the static lookup table (LUT) using the binary search and linear interpolation method. The fixed constant $\tau_{\mathrm{ref}}$ usually takes the average value of the measured pulse width $\tau_{m}$. For the convenience of calculation, $\tau_{\mathrm{ref}}$ is taken as 600 ns. The purpose of subtracting $\tau_{\mathrm{ref}}$ is to center the data, and its value does not affect the relationship between the distance deviation and pulse width. The pulse width difference Δτ and distance deviation ΔR were calculated. Detailed data is provided in Table 3.

The least squares method was applied to perform linear regression on the data points {Δτ,ΔR} and the compensation model was obtained as follows:

(20) $$\Delta R=-6.47\times 10^{-4}\cdot \Delta \tau -0.0932$$

### 4.3. Evaluation of Dynamic Compensation Algorithm

Equation (20) is the relationship between pulse width difference Δτ and distance deviation ΔR obtained by fitting using the least squares method. The fitting degree $R^{2}$ is 0.714, indicating a good linear relationship between Δτ and ΔR. To further verify the feasibility of the compensation algorithm, the random pulse widths were measured using the device under test, and the corresponding correction distances were calculated and compared with the actual distances.

The device under test measured pulse widths at nine random distances. The correction distance values were calculated using the dynamic compensation algorithm and compared with the actual distances, as shown in Table 4.

In nine random tests, the calculated correction distances of the device under test were in good agreement with the actual distances and the relative errors were basically controlled at a low level. The maximum relative error was 3.88% (corresponding to the actual distance of 2.32 m), the minimum relative error was 0.53% (corresponding to the actual distance of 1.88 m), and the average relative error was about 1.33%. These errors all meet the requirement of not exceeding 5%. This shows that the dynamic compensation algorithm has certain accuracy and practicability, and can be better used to convert the pulse width and distance of the device under test in this study.

## 5. Discussion

This study analyzes the variation in echo pulse width through the study of laser echo characteristics, and determines that there is an approximately linear negative correlation between the pulse width and detection distance. Therefore, a dynamic compensation method is proposed based on a dual-correction approach using a static lookup table and dynamic compensation. This method achieves distance correction by adding distance deviation compensation to the basic value from the static lookup table. The dynamic compensation system integrated with calibration and correction is designed and implemented. The feasibility of the dynamic compensation method is verified by testing.

This study validates the method using a single device, providing preliminary proof of the compensation mechanism’s effectiveness. Multiple devices need to be calibrated separately to establish their own exclusive compensation models. Future work will involve batch testing on multiple devices to further verify repeatability. The method relies on the consistency of the laser transmitter and receiver circuits. If key components (laser, photodiode, and amplifier) are changed, the LUT needs to be re-established because the approximate linear correlation range changes. Future work will explore the establishment of a LUT suitable for multiple scenarios and a wide range.

The testing range is limited by laboratory conditions but the current testing range (0.9–3.6 m) meets the requirements for this stage of validation, corresponding to the short distance scenarios of the target application. For longer distances, pulse broadening effects may become more pronounced, necessitating further adjustment of model parameters or the introduction of a segmented compensation strategy, which will be a focus of subsequent research. If nonlinearity occurs at very short or very long distances, the model can be extended by introducing a quadratic term, which represents a potential direction for method extension.

All tests were performed on static target to verify the basic performance of the compensation mechanism. Dynamic testing (e.g., moving target, vibrational environment) will be a key part of the next phase of field experiments. Experiments were conducted in a temperature-controlled laboratory using a white-colored paper target with relatively stable reflectivity. The standard sensor error (±0.5%) was incorporated into the overall error assessment. The influence of temperature and reflectivity variations on the compensation model will be systematically studied in the future. Future work could incorporate amplitude normalization or a dual-threshold method to reduce reflectivity dependence.

## 6. Conclusions

In this paper, a dynamic compensation method for laser ranging based on pulse width is proposed, which aims to improve the measurement accuracy of the laser ranging unit in an initiation device system, and ensure the accuracy and reliability of its fixed-distance initiation decision. The core of the method lies in the correction process based on the temporal feature of the echo pulse width. First, the static lookup table (LUT) is established for the device, providing a basic distance $R_{\mathrm{base}}$ from the original pulse width. Second, a dynamic device-specific compensation model is employed. This model uses the instantaneous pulse width to calculate a real-time compensation distance deviation value ΔR. Finally, the basic distance and distance deviation are added together to obtain the final distance. This dynamic compensation is applied to every single measurement. The specific work and results are as follows.

(1) The relationship between laser echo characteristics and the detection distance is analyzed. The laser echo signal attenuates greatly and the pulse width of the echo signal narrows significantly with an increase in the detection distance. When the detection distance increases from 0.75 m to 2.25 m, the maximum amplitude of the echo signal decreases from 5.9 V to 0.65 V, a decrease of approximately 89%. The pulse width decreases from 570 ns to 440 ns, a reduction of approximately 23%. Furthermore, within the middle range of 1 m to 5 m, the pulse width and detection distance exhibit an approximately linear negative correlation.

(2) A dynamic compensation method is proposed based on a dual-correction approach using a static lookup table and dynamic compensation, using the echo pulse width as the compensation information source for dynamic distance correction. The static lookup table for the pulse width and distance is established by using the high-precision standard distance sensor. By calibrating the device under test, its unique model of the relationship between the pulse width and distance deviation is established for dynamic compensation.

(3) The dynamic compensation system integrated with calibration and correction is designed and implemented. The feasibility of the dynamic compensation method is verified by testing. The relative error between the calculated correction distance and the actual distance is small, and the average relative error is about 1.33%.

## Figures and Tables

**Table 1:** Laser parameter settings.

Parameter	Value	Parameter	Value
$P_{0}$/W	25	$\eta_{\mathrm{atm}}$	0.9
*D*/m	0.01	$\eta_{\mathrm{sys}}$	0.8
ρ	0.9	*ℜ*/$V\cdot W^{-1}$	9
τ/ns	200	*M*	1000

**Table 2:** Static lookup table for pulse width and distance (LUT).

Distance $R_{\mathrm{std}}$/m	Pulse Width $\tau_{s}$/ns	Distance $R_{\mathrm{std}}$/m	Pulse Width $\tau_{s}$/ns
0.90	1020	2.30	645
1.00	975	2.40	634
1.10	931	2.50	623
1.20	884	2.60	612
1.30	845	2.70	595
1.40	814	2.80	574
1.50	791	2.90	555
1.60	763	3.00	540
1.70	740	3.10	521
1.80	718	3.20	506
1.90	697	3.30	494
2.00	681	3.40	481
2.10	668	3.50	467
2.20	656	3.60	453

**Table 3:** Measured and calculated data of the device under test.

Serial Number	Standard Distance $R_{m}$/m	Measured Pulse Width $\tau_{m}$/ns	Basic Distance $R_{\mathrm{base}}$/m	Pulse Width Difference Δτ/ns	Distance Deviation ΔR/m
1	1.0	886	1.196	286	−0.196
2	1.3	784	1.525	184	−0.225
3	1.5	739	1.705	139	−0.205
4	1.8	688	1.956	88	−0.156
5	2.0	662	2.150	62	−0.150
6	2.3	623	2.500	23	−0.200
7	2.5	602	2.659	2	−0.159
8	2.8	562	2.863	−38	−0.063
9	3.0	533	3.037	−67	−0.037
10	3.3	497	3.275	−103	0.0250
11	3.5	474	3.450	−126	0.0500

**Table 4:** Evaluation results of dynamic compensation algorithm.

Serial Number	Measured Pulse Width/ns	Calculated Correction Distance/m	Actual Distance/m	Relative Error/%
1	811	1.18	1.20	1.67
2	735	1.54	1.53	0.65
3	698	1.74	1.73	0.58
4	678	1.89	1.88	0.53
5	621	2.41	2.32	3.88
6	582	2.70	2.66	1.50
7	557	2.82	2.84	0.70
8	503	3.20	3.23	0.93
9	491	3.30	3.35	1.49

## Data Availability

All data generated or analyzed during this study are included in this published article.
